# Where they live, how they play: Neighborhood greenness and outdoor physical activity among preschoolers

**DOI:** 10.1186/1476-072X-10-66

**Published:** 2011-12-14

**Authors:** Diana S Grigsby-Toussaint, Sang-Hyun Chi, Barbara H Fiese

**Affiliations:** 1 Department of Kinesiology and Community Health, Division of Nutritional Sciences, University of Illinois at Urbana Champaign, USA; 2 Department of Geography, University of Illinois at Urbana Champaign, USA; 3 Family Resiliency Center, Department of Human and Community Development, University of Illinois at Urbana Champaign, USA

**Keywords:** Physical activity, preschoolers, children, neighborhoods, greenness

## Abstract

**Background:**

Emerging empirical evidence suggests exposure to "green" environments may encourage higher levels of physical activity among children. Few studies, however, have explored this association exclusively in pre-school aged children in the United States. We examined whether residing in neighborhoods with higher levels of greenness was associated with higher levels of outdoor physical activity among preschoolers. In addition, we also explored whether outdoor playing behaviors (e.g., active vs. quiet) were influenced by levels of neighborhood greenness independent of demographic and parental support factors.

**Results:**

Higher levels of neighborhood greenness as measured by the Normalized Difference Vegetation Index (NDVI) was associated with higher levels of outdoor playing time among preschool-aged children in our sample. Specifically, a one unit increase in neighborhood greenness increased a child's outdoor playing time by approximately 3 minutes. A dose-response relationship was observed between increasing levels of parental support for physical activity (e.g., time spent playing with children) and child outdoor physical activity (p < 0.01).

**Conclusions:**

Consistent with previous studies, neighborhood greenness influences physical activity behavior. However, for preschoolers, parental involvement may be more critical for improving physical activity levels.

## Background

Current rates of overweight and obesity among children in the United States (US) are a major public health concern [1-3]. Recent estimates from the National Health and Nutrition Examination Survey (NHANES) show that among 2-to-5 year olds, childhood obesity prevalence ranges from 7% among non-Hispanic White boys, 11% for non-Hispanic White and Mexican American girls, 14% for non-Hispanic Black boys and girls, and 17% for Mexican-American boys [4]. These rates represent an overall doubling of obesity in this age group over the last 30 years [5]. Given the increased risk for myriad health conditions (e.g., impaired glucose sensitivity and cardiovascular disease) that persist into adulthood due to childhood obesity, prevention efforts among pre-school aged children may be warranted [6,3,10].

The Council on Sports Medicine and Fitness of the American Academy of Pediatrics (AAP) recommends that preschool age children should take part in unstructured free play with an emphasis on running, tumbling, throwing, and catching [11]. However, physical activity (PA) in young children is influenced by the social context including presence of peers, the home environment, and access to green space or parks. A recent review of the literature concludes that there is large inter-individual variability in PA levels with some children being extremely active and others relatively sedentary [12]. Yet to be determined is whether the presence of green space contributes to this inter-individual variability.

Increasing children's exposure to built environments characterized by high levels of "greenness," broadly defined as vegetation such as trees and other plant life, has been shown to reduce obesity risk [13]. Both Bell et al., [14] and Liu et al., [15] found an inverse relationship between child overweight and residence in neighborhoods with dense vegetation. A suggested rationale for this association is that green neighborhoods are more likely to be both aesthetically pleasing and support more diverse types of physical activity, thus encouraging children to be more active outdoors [13,16]. This is supported by an evaluation of initiatives using landscape architecture to create green elementary school grounds in Canada, which found a 70% increase in light and moderate physical activity among children [16]. Time spent outdoors has also been shown to increase levels of physical activity among children, particularly among preschoolers [17,18].

Although emerging empirical evidence suggests an association between neighborhood greenness and reduced risk for overweight, few studies have been conducted exclusively among pre-school aged children. Preschool age children tend to engage in physical activity on a sporadic basis [19] and rely on their parents or caregivers to create opportunities for structured play and activity [20] in the US. Thus, future planning for interventions aimed at preschool age children must take the role of the family into account in contrast to school based interventions aimed at older children [19-21]. Furthermore, due to increasing rates of overweight in pre-school aged children and teenagers, it is also critical to develop sustainable preventive strategies to encourage physical activity at an earlier age [4,22,3]. Since outdoor play is important for physical activity among pre-school aged children, and neighborhood greenness encourages outdoor physical activity in older children (e.g., school age), we sought to, a) examine whether residing in neighborhoods with higher levels of greenness was associated with higher levels of outdoor physical activity among preschoolers, and, b) to explore whether outdoor playing behaviors (e.g., active vs. quiet) were influenced by levels of neighborhood greenness independent of demographic and parental support factors.

## Methods

### Sample

Preschoolers between ages 2-to-5 were recruited as part of the STRONG (**S**ynergistic **T**heory and **R**esearch on **O**besity and **N**utrition **G**roup) Kids Project, a three-wave study over five years which explores childhood obesity within a developmental ecological framework [23]. To ensure socio-economic and racial/ethnic diversity, an unequal probability sampling frame was used to identify licensed day care centers (n = 33) across five counties in Central Illinois. Beginning in January 2009, ninety-one percent (n = 30) of the centers permitted recruitment of children and their parents. A comprehensive self-report questionnaire designed to collect data on demographic characteristics, dietary and physical activity behaviors, and various aspects of parent-child relationships that moderate behaviors related to obesity risk among children was completed by enrolled parents [23]. Response rates among parents ranged from 60% to 95% across centers. Written informed consent was obtained from the parents of the children involved in this study. The data collection procedures for this study were approved by the Institutional Review Board at the University of Illinois at Urbana-Champaign.

Of the 424 surveys collected for wave one at the time of our analysis, 365 were included in our final sample. Fifty-nine cases were excluded due to incomplete addresses or missing data related to gender, race/ethnicity or education. Home addresses provided by parents were geocoded using a web-based geocoding batch program (http://www.gpsvisualizer.com/geocode) supplemented by manual matching. The use of the geocoding batch program allowed us to utilize the most recent commercial geographical database with a high level of accuracy [24].

### Dependent Variables

**Figure 1:**
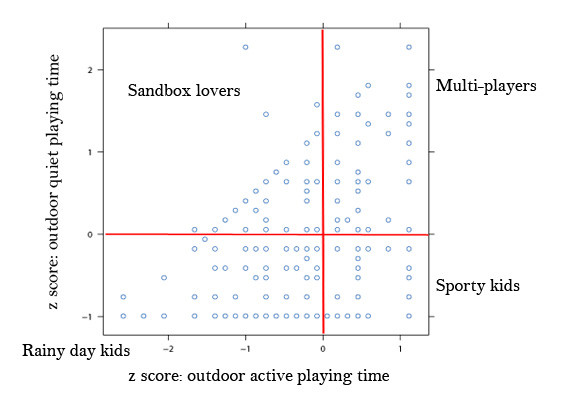
**Classification of physical activity behaviors**.

Two dependent variables were constructed for analyses. The first variable, *outdoor physical activity*, was based on questions included on the STRONG Kids survey from Burdette et al [18]. Parents were asked to report "how many minutes does your child spend" on each activity of indoor active playing, indoor quiet playing, outdoor active playing, and outdoor quiet playing on an "average WEEKDAY" as well as an "average WEEKEND DAY" (two separate questions). Parental responses to these questions have been shown to be highly correlated with measures of physical activity obtained by accelerometers [18]. Total outdoor playing time (in minutes) was calculated for each child to indicate outdoor physical activity. The second variable, categorized as *physical activity behaviors*, was constructed using z-scores of the amount of time children spent on active or quiet outdoor playing (Figure 1). Thus the z-score of each child represents the standardized value of time in active outdoor and quiet outdoor playing respectively. We plotted the z-scores on a two-dimensional plane where the × and Y axes represented active and quiet outdoor playing time, resulting in four "groups" of physical activity behaviors. The first group was labeled "*multi-players*" (n = 98) due to high z-scores for both outdoor active and quiet playing time. The second group (n = 49) was labeled "*sandbox lovers*" due to high scores on quiet outdoor playing time, but low scores on active outdoor playing time. The third group (n = 129) was labeled "*rainy day kids*" as they showed limited interest in any outdoor playing activities. The fourth group (n = 89), we labeled "*sporty kids*" due to much higher levels of outdoor active playing time, but limited outdoor quiet playing time.

### Neighborhood Greenness

To investigate the impact of neighborhood greenness on physical activity levels, satellite images of Landsat Thematic Mapper (TM) from the U.S Geological Survey (USGS) were used to calculate the normalized difference vegetation index (NDVI). The NDVI is the most widely used vegetation index and has been used in numerous studies to estimate vegetation biomass, greenness, and dominant species [25]. The NDVI calculation is based on the ratio between measured reflectivity in the red, and near infrared band, in satellite images because the chlorophyll pigment strongly absorbs radiation in the red band and is highly reflective in the near-infrared band [26]. Consequently, areas with dense vegetation show high values in the infrared band but low values in the red band. According to the NDVI formula, values vary from -1 to +1 [26]. The values close to 1 represent strong vegetation cover, while low values indicate rock and bare soil [15,25,26].

**Figure 2:**
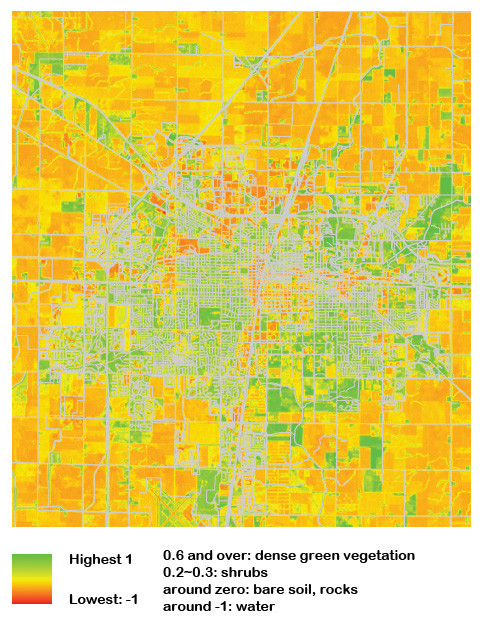
**Normalized Difference Vegetation Index (NDVI) of the study area**.

Although the NDVI is a useful standardized index, the presence of clouds may distort analyses [27]. To cope with this problem, the maximum-value composite method was used to acquire a cloud-free index [27,28]. In a nutshell, the maximum-value composite method is a process of overlaying multiple layers of the NDVI and extracting the maximal value since areas contaminated by cloud or cloud shade show exceptionally low values. For the composite method, 8 satellite images covering areas where the study participants resided were used. The most cloud-free images from July in each of the 4 consecutive years from 2007 to 2010 for the study area were selected. The Landsat TM represents a 30 by 30 meter area in one pixel. Thus a case that corresponds to a pixel would be assigned the mean NDVI value of a 900 square meter spatial unit. In order to account for the small size of the pixel, we enlarged the cell size to 90 by 90 by incorporating neighboring pixels. The size of the pixel was chosen for two reasons. First, despite recent developments in geo-mapping technology and satellite images, it is still possible to have geocoded coordinates differ slightly from actual locations [29]. Consequently, the resized pixel allowed us to deal with accuracy issues that may arise from the geocoding process. Using the 90 by 90 meter configuration allowed us to account for the possibility that a participant's home would fall into a neighboring pixel - something we could not account for as well using the30 by 30 meter configuration. Second, given extant research indicating that children usually play close to their homes [30], 8100 square meters should encompass the size of a child's own home, in addition to the immediate neighborhood. Consistent with previous studies [14,15] we also scaled the NDVI by a factor of 10 to facilitate easier interpretation of increases in the NDVI. The distribution of NDVI is similar to that of the normal distribution. In our study, the minimum NDVI value was 1.06 and the maximum was 7.31 (Figure 2). The first (25%) quartile corresponds to 3.83, and the third is 5.3.

### Control Variables

Parental self-reported data from the STRONG Kids survey were used to control for the influence of socio-demographic characteristics on our outcomes of interest. Specifically, race/ethnicity, White, compared to all other groups combined (non-White), and gender, were included as binary variables in our models. Non-Whites and females were used as our reference categories Due to the limited sample size of each specific racial/ethnic minority group (e.g., African-American, Asian), to ensure power in our analyses, we collapsed all racial/ethnic minority groups and compared them to Whites. Parental education was categorized as less than a college degree (*reference category*), college degree, or post-graduate degree. In addition, parental support for physical activity was measured using the following questions from the SPARK survey [31], "During the past week, how often has an adult in your family done a physical activity or played sports with your child?," and "During the past week, how often has an adult in your family transported your child to a place where he or she can do physical activities or play sports?" The responses were "none", "once," "a few times," and "often." Levels of support were then categorized as low (none), medium (once or a few times), and high (often).

### Statistical Analysis

Stata version 11 [32] was used to conduct descriptive and multivariate regression analysis. To explore the influence of neighborhood greenness on total outdoor playing time, a linear regression model was run controlling for race/ethnicity, parental education, and parental support for physical activity. Multinomial regression was used to examine the association between neighborhood greenness and physical activity patterns among children, also controlling for race/ethnicity, parental education, and parental support for physical activity. Children categorized as "*rainy day kids*" were used as the reference for this analysis. Descriptive statistics were also summarized for all study variables.

## Results

On average, the children in our sample engaged in 60 minutes of outdoor playing time per day. Based on guidelines from the National Association for Sport and Physical Activity (NASPE), most children in the sample are meeting the suggested 60 minutes of physical activity each day [33].

Table 1 summarizes the characteristics of the study sample by total outdoor physical activity. Boys were significantly more likely to engage in active outdoor physical activity compared to girls (p < 0.01). A dose response relationship was also observed between higher levels of parental support for physical activity (e.g., time spent playing with children) and higher levels of child outdoor physical activity (p < 0.01, and 0.01 respectively). While there were no significant differences observed between levels of parental education and outdoor physical activity, White children were more likely to engage in outdoor physical activity compared to non-White children (p = 0.01).

**Table 1:** Characteristics of the study population by outdoor playing time (n = 365)

Variables	Cases /mean (S.D)	percent		Outdoor playing time ^a^		
			
			active	*P*-value ^b^	quiet	*P*-value
**NDVI**	4.5 (1.1)					
**Gender**						
Girls	175	48%	38.5	< 0.01	18.4	0.85
Boys	190	52%	45.2		18.1	
**Race/ethnicity**						
non-white	142	39%	39.4	0.01	15.5	0.02
white	223	61%	43.6		20.0	
**Education**						
less than college degree	141	39%	42.4	0.69	19.3	0.40
college degree or higher	224	61%	41.7		17.6	
**Parent's BMI**	26.6 (6.5)					
**Parental time spent** **playing with child**						
Low	66	18%	35.0	< 0.01	15.0	0.06
Medium	153	42%	40.6		17.2	
High	146	40%	46.5		20.9	
**Parental time spent** **transporting child to PA**						
Low	184	50%	39.0	0.01	13.6	< 0.01
Medium	112	31%	44.3		21.1	
High	69	19%	46.3		26.0	

_a _unit: minutes per day

_b _*P*-value of two-way ANOVA test to test the difference of means between categories.

Table 2 summarizes the characteristics of the study sample by physical activity behaviors. The mean NDVI was highest for children categorized as *multi-players*. Boys, White children, and children whose parents often spent time playing with them were more likely to be categorized as *multi-players*. Girls, children whose parents had less than a college degree, or spent limited time transporting them to places to engage in physical activity were more likely to be placed in the *rainy day *category. Non-white children typically exhibited physical activity behaviors of the *rainy day *category, while White children were most likely to fall into the *multi-player *category. As the amount of time parents spent playing with their children increased, the more time children spent engaged in outdoor physical activity (i.e., exhibiting *multi-player *or *sporty kids *physical activity patterns).

**Table 2:** Characteristics of the study population by physical activity behaviors (n = 365)

	Multi-players	Sandbox lovers	Rainy day kids	Sporty kids	p- value^a^
**Outdoor playing time (minutes per day)**					
Active outdoor playing	55.6	32.2	26.3	55.0	< 0.001
Quiet outdoor playing	41.0	27.7	5.60	6.40	< 0.001
**NDVI**	4.80	4.60	4.40	4.40	0.054
**Gender**					0.014
Girls	44	30	69	32	
Boys	54	19	60	57	
**Race/ethnicity**					0.046
non-white	28	21	60	33	
white	70	28	69	56	
**parent's education**					0.400
less than college degree	40	14	54	33	
college degree and over	58	35	75	56	
**Parent's BMI**	26.4	25.5	27.6	26.2	0.194
**Parents' intervention: playing with kid**					0.002
Low	12	13	29	12	
Middle	36	19	65	33	
High	50	17	35	44	
**Parents' intervention: transporting child to PA^b^**					0.002
Low	31	26	79	48	
Middle	39	15	32	26	
High	28	8	18	15	

_a _we used ANOVA for rate variables and chi-square for binary variables

_b _PA = physical activity

Table 3 summarizes the results of the linear regression model with total outdoor physical activity as the dependent variable. Higher levels of neighborhood vegetation as measured by the NDVI was associated with higher levels of physical activity (*b *= 2.82, 95% CI = 0.21, 5.43) after controlling for race/ethnicity, gender, parental education and BMI, as well as parental support for physical activity. Specifically, a one unit increase in neighborhood greenness increases a child's outdoor physical activity time by approximately 3 minutes per day in our sample. White children, as well as children whose parents spent considerable time playing with them or transporting them to opportunities for physical activity had higher levels of total outdoor physical activity. Higher levels of parental BMI was found to have a negative influence on outdoor physical activity, but this relationship was only found to be marginally significant.

**Table 3:** Linear regression of neighborhood greenness on outdoor physical activity

	B (95% CI)	Beta	P-value
**Neighborhood greenness**			
NDVI	2.82 (0.21, 5.43)	.107	.034
**Gender (ref: female)**			
Male	4.55 (-1.30, 10.40)	.078	.127
**Race/ethnicity (ref: non-white)**			
white	6.68 (0.78, 12.50)	.112	.027
**Parental education** **(ref: less than college degree)**			
College degree and more	-4.64 (-10.76, 1.46)	-.077	.135
**Parental weight status ^b^**			
Parent's BMI	-0.42 (-0.87, 0.03)	-.094	.070
**Parental support:** **Time spent playing with child (ref: low)**			
Medium	4.62 (-3.46, 12.72)	.078	.262
High	9.54 (0.98, 18.10)	.160	.029
**Parental support:** **Transporting child to PA^a ^(ref: low)**			
Medium	10.64 (4.01, 17.26)	.168	.002
High	16.48 (8.52, 24.44)	.222	< 0.001

_a _PA = Physical activity

_b _parent completing the survey, usually mothers

Table 4 summarizes the results of the multinomial logistic regression model with physical activity behaviors as the dependent variable. (Sandbox lovers were excluded from the analysis due to the small sample size). *Rainy day kids *were used as the reference for all models. Higher levels of neighborhood greenness was associated with higher odds of exhibiting *multi-player *physical activity behavior (OR = 1.32, 95% CI = 1.02, 1.71). *Multi-player *physical activity behaviors were more likely to be exhibited by White children (OR = 1.87, 95% CI = 1.04, 3.38), and children whose parents spent a considerable amount of time transporting them to opportunities for physical activity. *Sporty kids *were more than twice as likely (OR = 2.36, 95% CI = 1.00, 5.58) to have parents spend considerable time playing with them, compared to *rainy day *kids. *Sporty kids *were also more likely to be male, compared to *rainy day *kids, but this association was only marginally significant.

**Table 4:** Multinomial logistic regression of neighborhood greenness and physical activity behaviors of children (N = 365)^a, b^

	Multi-players		Sporty kids	
	**OR^d ^(95% CI)**	**p-value**	**OR^d ^(95% CI)**	**p-value**
**Neighborhood greenness**				
NDVI	**1.32 (1.02, 1.71)**	**0.037**	1.02 (0.79, 1.32)	0.867
**Gender **(ref: female)				
Male	1.27 (0.71, 2.26)	0.419	1.72 (0.96, 3.09)	0.068
**Race/ethnicity **(ref: non-white)				
white	**1.87 (1.04, 3.38)**	**0.038**	1.42 (0.80, 2.53)	0.227
**Parental overweight**				
Parent's BMI	0.98 (0.93, 1.02)	0.264	0.97 (0.93, 1.01)	0.192
**Parental support:**				
**Time spent playing with kid **(ref: low)				
Medium	1.01 (0.44, 2.32)	0.981	1.05 (0.47, 2.39)	0.899
High	2.13 (0.89, 5.09)	0.089	**2.36 (1.00, 5.58)**	**0.049**
**Parental support:**				
**transporting child to PA^c ^**(ref: low)				
Medium	**2.80 (1.45, 5.40)**	**0.002**	1.30 (0.67, 2.52)	0.436
High	**3.06 (1.41, 6.67)**	**0.005**	1.02 (0.45, 2.33)	0.958

_a _ref: *Rainy Day Kids*

_b _*Sandbox lovers *category was excluded due to small sample size

_c _PA = physical activity

_d_OR = odds ratio

## Discussion

Our study sought to build upon studies examining aspects of the natural environment that influence childhood obesity risk [13-16]. Consistent with previous studies [14-16], we found that exposure to higher levels of neighborhood greenness may reduce the risk for childhood obesity due to increased physical activity. Specifically, after controlling for socio-demographic and parental support factors, children residing in neighborhoods with higher levels of neighborhood greenness were more likely to engage in outdoor physical activity. To further explore this relationship, we examined whether physical activity behavior (i.e., total outdoor playing time, active vs. quiet play) was influenced by neighborhood greenness. Children living in neighborhoods with the highest levels of neighborhood greenness were more likely to engage in outdoor physical activity (*multi-players*), and engaged in similar bouts of active and quiet outdoor play. This suggests that exposure to greener neighborhoods encourages children to spend more time outdoors where they may reap both physiological and cognitive benefits [13,34]. Conversely, children with the lowest levels of neighborhood greenness were least likely to spend time playing outdoors, engaging in active or quiet play (*rainy day kids*). While neighborhood greenness influenced levels of physical activity among these preschool-aged children, as Cleland et al [35] observed, parental support factors such as engaging in physical activity with children also plays a role. This may explain why *sporty kids *were more likely to engage in much more active outdoor physical activity compared to *rainy day kids*, although both groups lived in areas with similar levels of neighborhood greenness (Table 2). Moreover, although *sandbox lovers *had higher levels of neighborhood greenness than *sporty kids*, their parents were least likely to transport them to opportunities for physical activity (Table 2). It is important to note, however, that perhaps the personality of some of the children, such as the *rainy day kids*, with the tendency to engage in limited outdoor activity, may also influence parents to spend less time transporting them to opportunities for physical activity.

Since the research literature suggests that boys are typically more active than girls between pre-school age and adolescence, we were not surprised to find that boys tended to engage in more physical activity, compared to girls in our sample (Tables 1 and 2) [31,36,37]. Socialization by gender, where boys may be encouraged to engage in more physical activity may explain this difference.

While our findings support previous studies exploring the influence of neighborhood greenness on childhood obesity risk, there are some limitations. Our study was cross-sectional, and focused only on levels of physical activity, rather than BMI. Moreover, our measure of physical activity relied on parental reports, rather than objectively measured data collected from accelerometers. Notwithstanding, the quantification of physical activity among preschoolers using accelerometers has been wrought with challenges due to the inability to account for differences in motor and cognitive development that may influence movement patterns [38-40]. While accelerometers seem to be far more effective measuring physical activity among school-age children, standardized methods are still being developed for preschoolers [38].

Our sample size may have also been too small to give us more robust estimates, as indicated by the wide confidence intervals for some of the results of our regression analyses. Our omission of certain measures of the neighborhood environment may have also influenced our estimates, such as neighborhood *walkability*, which has been shown to influence overweight among pre-school aged children [41]. We also did not control for neighborhood measures of socioeconomic status, which have been shown to influence childhood overweight, independent of neighborhood greenness [14]. When we included measures of neighborhood SES, however, they were highly correlated with NDVI, so we did not include them in our models to avoid multi-collinearity. Interestingly, when we ran our models using only measures of neighborhood SES to determine whether the NDVI was strictly serving as a proxy for SES, we did not find the same relationship with levels of physical activity. Specifically, while higher levels of neighborhood greenness were associated with higher levels of physical activity, the same did not hold true for higher levels of neighborhood SES. This observation may be due to the fact that the study area encompasses micro-urban and semi-rural areas, where neighborhood SES may be less important for access to greenspace, compared to many urban environments in the US. We also did not account for seasonality based on the time of the year that parents completed the survey. A study by Finn et al. [42], however, found that seasonality did not influence the total physical activity time of pre-school aged children. In addition, our own analysis (not shown) did not indicate that seasonality influenced levels of physical activity reported by parents.

Another limitation is the use of the area around the home address as the spatial unit for measuring neighborhood greenness. However, we believe that using the home address of pre-school aged children in this sample was the best spatial proxy for measuring the influence of neighborhood greenness on physical activity for several reasons. First, there is some evidence [30] to suggest that children usually play close to where they live. As such, we attempted to account for areas close to where children live that would encompass nearby playspaces in addition to their backyard, for example. Of course, it is still possible that parents could take their children to places outside of their immediate neighborhoods to engage in active play. Given what the authors know of the study area, however, many families of young children do in fact regularly utilize their neighborhood playspaces. Second, approximately 20% of children in our sample did not attend day care every day, and only 30% of parents reported having their children in day care 40 hours a week. As such, we could only assume that the children were also spending quite a bit of time at their home address. Third, parents were asked to separately report on weekend and weekday physical activity, thus the home address is the only spatial unit that is consistent across all 7 days of the week. Given what we knew of day care attendance in our sample, and park/playspace utilization in the study area, we believe that we used the best spatial proxy for measuring neighborhood greenness based on our study design and resources. We do concede, however, that the use of GPS to actually track the children during the day, or using an activity space log would have more accurately captured the play spaces of the children in our sample.

Another issue that we also explored was the potential cluster effect of day care centers. However, our preliminary analyses showed an intraclass correlation of less than .01. As such we felt confident that the relationships observed in our study were not overestimated.

Despite these limitations, our study also has several strengths. We used an objective measure of neighborhood greenness, and examined both total outdoor playing time and physical activity patterns among children. Our study is also one of the first to examine the influence of the natural environment on childhood obesity risk that focuses exclusively on pre-school aged children and accounts for physical activity behaviors, socio-demographic and parental support factors. By focusing exclusively on pre-school aged children, this allows us to tailor efforts on a critical developmental stage for targeting interventions.

## Conclusion

Access to greenspace has been associated with several aspects of human health, including improved mental health and higher levels of physical activity among children and adults [13,16,43]. Although largely indirect, evidence from several studies suggests that individuals residing in neighborhoods with access to greenspace are more likely to be imbued with an increased sense of community [43]. Consequently, parents of children residing in these neighborhoods may not only encourage their children to engage in more physical activity, but also ensure that neighborhoods are adequately policed and maintained to facilitate active play. This may explain our results, where higher levels of neighborhood greenness were associated with increased levels of outdoor physical activity among preschoolers. However, parental co-participation in physical activity or transportation to opportunities to engage in physical activity modified the relationship. Our findings further support initiatives to encourage exposure to accessible outdoor green spaces for preschool aged children and their families.

## Competing interests

The authors declare that they have no competing interests.

## Authors' contributions

DGT conceptualized the study, supervised the data analysis, and drafted the manuscript. SHC performed data analysis, contributed to the interpretation of results, and the revision of the manuscript. BHF contributed to data acquisition, interpretation of results, and revision of the manuscript. All authors read and approved the final manuscript.
